# Highlighting the Use of the Hepatoprotective Nutritional Supplements among Patients with Chronic Diseases

**DOI:** 10.3390/healthcare11192685

**Published:** 2023-10-06

**Authors:** Monica Foghis, Delia Mirela Tit, Simona Gabriela Bungau, Timea Claudia Ghitea, Csaba Robert Pallag, Andreea Monica Foghis, Tapan Behl, Cristian Bustea, Annamaria Pallag

**Affiliations:** 1 Doctoral School of Biomedical Sciences, Faculty of Medicine and Pharmacy, University of Oradea, 410073 Oradea, Romania; monica.foghis@student.uoradea.ro (M.F.); apallag@uoradea.ro (A.P.); 2 Department of Pharmacy, Faculty of Medicine and Pharmacy, University of Oradea, 410028 Oradea, Romania; timea.ghitea@csud.uoradea.ro; 3 MSc International Economy and Business Program of Study, Corvinus University of Budapest, 1093 Budapest, Hungary; csaba.pallag@stud.uni-corvinus.hu; 4 Medicine Program of Study, Faculty of Medicine and Pharmacy, University of Oradea, 410073 Oradea, Romania; foghis.andreeamonica@student.uoradea.ro; 5 School of Health Sciences & Technology, University of Petroleum and Energy Studies, Dehradun 248007, India; tapan.behl@ddn.upes.ac.in; 6 Department of Surgery, Oradea County Emergency Clinical Hospital, 410169 Oradea, Romania; cristian.bustea@uoradea.ro

**Keywords:** chronic diseases, hepatoprotective supplements, survey, nutritional status

## Abstract

Cross-sectional studies, while not considered glamorous endeavors, are firmly anchored in data and statistics, providing essential insights about public health. The aim of the study is to see the frequency of hepatoprotective (HP) nutritional supplement consumption among patients with chronic diseases (other than chronic liver disorders) and analyzes the habits related to the consumption of nutritional supplements among these patients. A total of 954 patients, seeking medical prescriptions for chronic diseases under various payment arrangements (compensated, gratuity, or full payment) were carefully selected over a 12-month period from four private pharmaceutical facilities. We examined the frequency of HP consumption in relation with a number of prescribed medications for chronic conditions. All these patients were invited to complete a questionnaire about their supplement consumption habits and were provided the option to participate in a nutritional status assessment. One hundred ninety-five patients consented to participate in the survey, and 65 patients agreed to undergo a nutritional status evaluation. Of the 954 patients, 77.2% incorporate HP into their regimen. The most frequent consumption (83.33%) was recorded in a group with seven drugs, followed by a group with three drugs (82.84%). Women have a higher usage rate of HP (80.58%; 444 from 551) compared to men (62.60%; 293 from 383), and most of the patients (59.5%) used extracts of Silybum marianum L. In the survey, 64.61% of participants were using supplements, with most (59.52%) consuming HP. Only 32.54% of patients rely on recommendations from healthcare professionals. Of the patients who use supplements, 55.56% reported improvements in their health status. Furthermore, patients who integrate supplements into their daily routine tend to achieve better overall nutritional status.

## 1. Introduction

Chronic diseases place a significant burden on global health [1]. According to the World Health Organization (WHO), chronic diseases are characterized by certain key attributes, including their long-lasting nature, resulting residual disabilities, non-reversible pathological changes as their underlying cause, the need for specialized patient training for rehabilitation, or extended periods of supervision, observation, or care [2,3].

In developed countries, there is a high prevalence of individuals suffering from multiple chronic conditions, known as multi-morbidity [4], which tends to increase with age [4,5]. The management of chronic diseases poses a substantial challenge to healthcare systems worldwide, as these systems have traditionally focused on addressing acute episodes rather than delivering organized, long-term care for patients dealing with chronic conditions [6]. A key characteristic of chronic diseases is the necessity for ongoing supervision, care, and observation. Primary care, which is defined by qualities such as continuity, coordination, and addressing complexity, is well-suited to handle the management of chronic conditions [7]. There is growing evidence underscoring the importance of reorienting health policies and healthcare systems toward chronic care, with an emphasis on strengthening primary care [8]. Countries with robust primary care systems typically achieve better health outcomes at a lower cost [9].

In the realm of liver disease, experts have extensively explored the topic of drug-induced liver injury over the past three decades [10,11,12].

Chronic drug use is known to cause persistent damage, where the liver’s ability to regenerate eventually becomes dysfunctional, leading to liver scarring and cirrhosis. Despite recent therapeutic advances and significant development in modern medicine, liver diseases remain a global health problem, affecting over 10% of the global population [13]. Chronic hepatic disorders remain a significant concern globally [14] due to the high mortality rate associated with liver cirrhosis in Western nations.

Nutritional supplements are products designed to provide essential nutrients that may be lacking in sufficient quantities in a person’s diet. Natural hepatoprotective products derived from herbs, such as *Silybum marianum* L. Gaertn., *Cynara scolymus* L., *Cichorium intybus* L., *Capparis spinosa* L., *Glycyrrhiza glabra* L., *Ganoderma lucidum* Karst., *Chlorella* ssp., and other species containing various bioactive compounds, can have a significant positive impact on liver metabolic parameters in patients with chronic diseases [14,15,16]. These naturally hepatoprotective substances, with different structures, but sharing the same therapeutic activity, can act as active agents in various chronic diseases. Several phyto molecules, including flavonoids, alkaloids, glycosides, and saponins obtained from various plant sources, have been reported as potent hepatoprotective agents [17].

Studies have shown beneficial effects of certain isolated bioactive compounds, such as polyphenolic compounds like curcumin [18], resveratrol [19], and quercetin [20], on metabolic parameters in these patients. Similarly, other natural products like grapes [21], green tea, orange juice [22], hibiscus [23], aloe vera [24], wild bitter, berberine [25], barberry [26], and withaferin A [27,28] have demonstrated similar effects on metabolism.

Both in ancient and modern phytotherapy, extremely versatile and valuable plants with hepatoprotective properties are used and studied continuously. Examples of such plants include milk thistle (*Silybum marianum* L. Gaertn.), artichoke (*Cynara cardunculus* L.), and chicory (*Cichorium intybus* L.) [29]. These plants have been specifically chosen due to the global concern surrounding liver diseases, as all parts of these plants have potential applications. Artichoke and chicory are commonly consumed as foods or dietary supplements, with less frequent use as phytomedicines [30]. Milk thistle, renowned for its hepatoprotective properties, is recognized for its ability to support liver functions, and dietary supplements and phytomedicines derived from milk thistle fruits/seeds are considered effective remedies in this regard [31]. Currently, dietary supplements based on milk thistle rank among the top 40 best-selling herbal supplements [32]. Over the past decade, there has been a significant rise in the demand for phytomedicines and dietary supplements with hepatoprotective properties. This surge can be attributed to the abundance of information available on their therapeutic significance.

Bioactive compounds can be used as strategies for the prevention and treatment of metabolic disorders, specific to many chronic conditions, by improving the inflammatory state and other comorbidities, such as obesity, dyslipidemias, and cardiovascular diseases [33].

The aim of the study is to see the frequency of HP consumption as adjuvant treatments among patients with chronic diseases (other than chronic liver disorders) and analyzes the habits related to the consumption of nutritional supplements among these patients. Additionally, the differences in nutritional status, assessed by measuring body composition between consumers and non-consumers of nutritional supplements, were evaluated.

## 2. Materials and Methods

### 2.1. Study Design

In the first part of this cross-sectional study, we analyzed frequency of HP consumption among 954 patients with chronic diseases from Bihor County, Romania, in addition to prescribed medications for their conditions. Data from four pharmacies in the city of Oradea, Bihor County, Romania were analyzed over 12 months (from October 2021 to October 2022). Patients with medical prescriptions for chronic diseases were recruited based on the diagnosis provided by the current doctor. The selection criteria were as follows: age over 18 years, expressing the agreement to participate in the study, the presence of one or more chronic diseases, and the prescribed treatment containing one or more allopathic drugs. The exclusion criteria were the patient’s refusal to participate in the study, the presence of acute diseases, the presence of chronic liver diseases, and psychiatric and oncologic disorders. Having chronic diseases, patients are treated with one or more drugs for a long time without interruption. The 954 patients selected and included in the analysis were categorized into seven groups according to the number of drugs prescribed for the chronic disease/diseases (from Group 1 with one medicine, to Group 7 with seven or more medicines) (Table 1). Data processing and statistical analysis were conducted to examine frequency in use of HP across the study groups, as well as the types of nutritional supplements administered.

All patients included in the analysis were invited to complete a questionnaire that evaluates the habits related to the consumption of food supplements and to participate in a nutritional assessment. One hundred ninety-five patients consented to participate in the survey, and 65 patients agreed to undergo a nutritional status evaluation.

### 2.2. Evaluation of Supplement Usage Habits

An examination tool was developed as a questionnaire of seven questions, both open-ended and closed-ended, in the Romanian language (translated as Supplementary Material S1).

The package of questions, as well as the answer options, was chosen and established following consultations with professionally experienced pharmacists (in the community pharmacy), for more than 5 years. Items were developed for identifying the habits regarding the consumption of food supplements among the population with chronic diseases. The questions were formulated in such a way as to be as simple as possible and easy to understand to result in a sufficiently relevant questionnaire, but also to be as short as possible.

The first section of the questionnaire establishes the socio-demographic criteria of the participants, the habit of consuming food supplements or not, the purpose of their use, the type of supplements consumed, and the way in which the used supplement was chosen. The second section establishes whether the recommended doses are respected, the duration of use, and the self-assessed impact on health.

A number of 20 patients with chronic diseases answered the questions from the initial draft of the questionnaire (pilot test). In the final form, there were minor editorial changes, and the items were supported by the initial draft. Experts from the College of Pharmacists from Bihor County (the representative forum of pharmacists for Bihor County, which was also the county professional organization of pharmacists and a sub-branch of the College of Pharmacists from Romania) evaluated the final version of the questionnaire for content validity. After that, face-to-face interview with trained interviewers was used to collect the data from the 195 patients who participated in the survey. Internal consistency of the questionnaire was estimated using the coefficient Cronbach’s alpha [34]. The average value of Cronbach’s Alpha test for the seven questionnaire items was 0.886, falling into the “good” category.

### 2.3. Assessment of Nutritional Status

Evaluation of the nutritional status was conducted using the Tanita MC 780 MA body bioelectrical impedance analyzer (BIA) from Tokyo, Japan [35,36]. The collected data were evaluated using the GMON 3.4.1 medical software from Chemnitz, Germany.

BIA body analyzers, approved by the World Public Health Nutrition Association (WPHNA), were employed to determine body composition with high precision, including measurements of BMI (body mass index), fat mass, muscle mass, sarcopenic index, and total body water (TBW) hydration status. The margin of error for these measurements was 0.1 kg.

Regarding BMI, the following classification was used:

- 18.49 or less—Underweight;
- 18.50 to 24.99—Normal weight;
- 25.00 to 29.99—Overweight;
- 30.00 to 34.99—Obesity (degree I);
- 35.00 to 39.99—Obesity (degree II);
- 40.00 or more—Obesity (degree III) or morbid obesity.

Both men and women were included in these categories.

For fat mass, normal range for women is between 23% and 34%, and for men, it is between 13% and 25% (marked as 1 in our study). If the subject had a fat mass below the normal range, it was marked as “0” in both cases. In men, a range between 25% and 30% is considered excessive (marked as 2), and over 30% is classified as very high (marked as 3). In women, the excessive range is between 34% and 40% (marked as 2), and over 40% is classified as very high fat mass (marked as 3).

According to body analyzer, the normal ranges for muscle mass are as follows:

- Ages < 65: 75–89% for men, 63–75.5% for women.
- Ages 65–70: 73–86% for men, 62–73.5% for women.
- Ages > 70: 70–84% for men, 60–72.5% for women.

In our study, these intervals were scored as 1. Individuals with muscle mass below the normal range were marked as 0, while those with muscle mass above the normal limit were marked as 2.

For the sarcopenic index, normal values for women should be above 5.85, and for men, it was above 7.00. High values of sarcopenic index are associated with the infiltration of fats between actin and myosin, which can block insulin receptors and correlate with metabolic syndrome.

Water mass, expressed as total body water (TBW), is recommended to be between 45% and 60% for normal limits (marked as 1 in our study). These values are modified according to age and sex. The ranges are as follows:

- Ages < 65: Males 52–66% (average: 59%), Females 49–63% (average: 56%).
- Ages 65–70: Males 43–73% (average: 59%), Females 41–60% (average: 50%).
- Ages > 70: Males 47–67% (average: 56%), Females 39–57% (average: 47%).

Changes in total water volume are not directly related to water consumption but can be influenced by various factors such as inflammation, kidney disease, and unhealthy levels of fat mass (obesity).

In order to carry out the research included in this study, the approval (with the reference number CEFMF/19 dated 26 February 2021) of the Research Ethics Committee of the Faculty of Medicine and Pharmacy, University of Oradea, was obtained.

### 2.4. Statistical Analyses

The collected data were analyzed using the statistical software SPSS 20 (New York, NY, USA) through statistics such as ANOVA, Post-Hoc analysis, chi-square test, and inferential statistics (Student *t*-test). The three research groups were compared using the Bonferroni test.

## 3. Results

### 3.1. Hepatoprotective Nutritional Supplements Used

The average age of participants was 71.22 ± 15.47 years. Among them, 383 were male (40.1%) and 571 were female (59.9%). A majority of 783 individuals (82.1%) came from urban areas, while 171 (17.9%) were from rural areas. Out of the 954 total participants, 737 used HP in addition to their allopathic treatment.

Almost 60% of them (58.9%, N = 434) fell into the groups with two and three prescribed medications, in equal proportions, followed by Groups 4, 5, and 6. At the opposite pole, with the fewest patients, Groups 7 (5%, N = 48) and 1 (6.5%, N = 62) are found.

**Figure 1:**
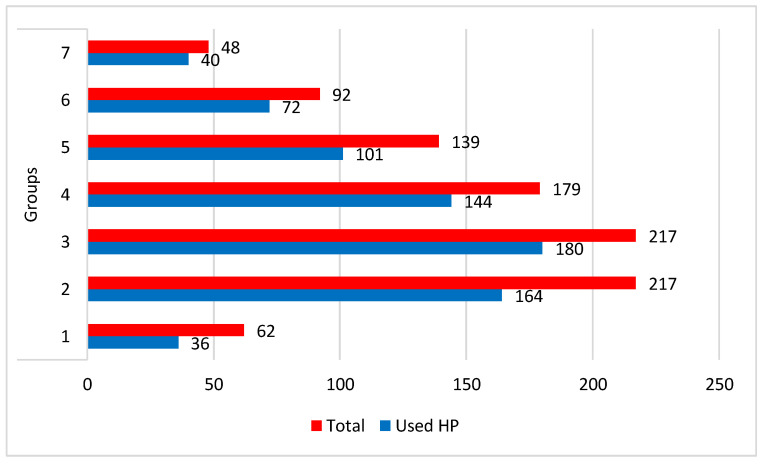
Distribution of the use of hepatoprotective nutritional supplements (HP) in the seven investigated groups; 1–7, number of group/medicines for chronic conditions.

The frequency of HP consumption varied depending on the number of prescribed drugs. Compared to the number of patients in each group, the most frequent consumption (83.33%, N = 40/48) was recorded in Group 7, followed by Group 3 (82.84%, N = 180/217) and Group 4 (80.44%, N = 144/179), while the lowest usage was recorded in Group 1 (58%, N = 36/62) (Figure 1).

**Figure 2:**
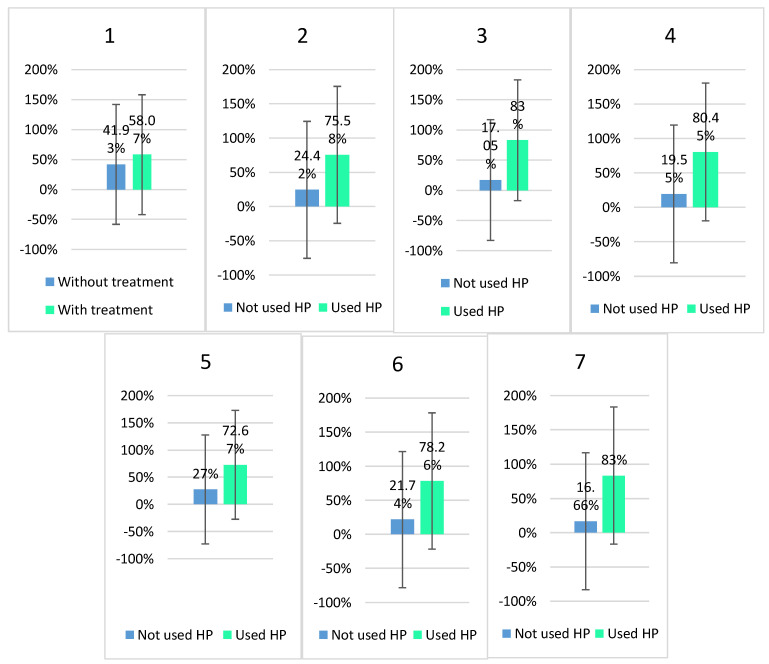
Percentage breakdown of hepatoprotective nutritional supplement (HP) consumption. 1–7—number of group/medicines for chronic conditions.

The percentage breakdown of HP consumption among studied patients among different groups are presented in Figure 2. Among those who did use HP, the following preferences were recorded: *Silybum marianum* L. Gaertn. extracts, standardized silymarin, essential phospholipids, L-arginine, artichoke extracts, *Armoracia rusticana* L. and silymarin extracts, *Cichorium intybus* L. extracts, choline citrate with silymarin extract, alpha-lipoic acid, combination of lecithin, with silymarin, etc. The patients can be divided into three categories: patients who do not use supplements, and patients using supplements based on *Silybum marianum* L. Gaertn., patients using another type of supplement, based on *Cichorium intybus* L., *Armoracia rusticana* L., *Carum carvi* L., *Curcuma longa* L., *Piper nigrum* L., *Ganoderma lucidum* Kanst., *Chlorella* spp., *Spirulina* spp., soybean seeds, capers, Indian currant extracts, essential phospholipids, L-arginine.

**Figure 3:**
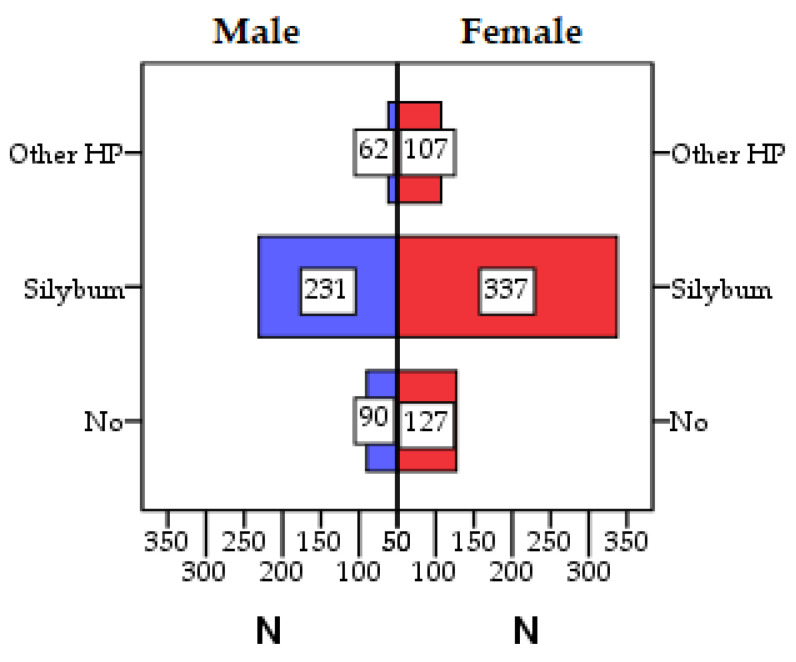
The use of hepatoprotective nutritional supplements (HP) according to gender. No—patients who do not use HP; Silybum—patients using HP based on *Silybum marianum* L. Gaertn; Other HP—patients using another type of supplement based on *Cichorium intybus* L., *Armoracia rusticana* L., *Carum carvi* L., *Curcuma longa* L., *Piper nigrum* L., *Capparis spinosa* L., *Glycyrrhiza glabra* L., *Ganoderma lucidum* Kanst., *Chlorella* spp., *Spirulina* spp., soybean seeds, Indian currant extracts, essential phospholipids, L-arginine.

The analysis of the use of supplements according to gender indicates that women have a higher usage rate of HP (80.58%; 444 from 551) compared to men (62.60%; 293 from 383) (Figure 3).

Table 2 displays the distribution of HP consumers across different groups, categorized by the type of hepatoprotective active substance. Notably, most of the patients (59.5%) used extracts of *Silybum marianum* L. Gaertn while 17.7% of patients opted for a different hepatoprotective substances.

### 3.2. Supplement Usage Habits

One hundred ninety-five patients with an average age of 60.26 ± 14.12 completed the questionnaire. The socio-demographic and clinical characteristics of the participants are presented in Table 3. Dietary supplement consumption habits were analyzed by age groups (<65, 65–70, and >70 years old). Most of the respondents were women (75.38%), they were under 65 years old (49.23%), and they came from the urban environment.

One hundred twenty-six of the 195 patients (64.61%) declared that they consume nutritional supplements.

In the group of patients under the age of 65 years old, 32% of the respondents used nutritional supplements, which was significantly more than in the 65–70 (21.5) or >70 (10.8) years old patients’ category. Hepatoprotective supplements were the most frequently used (75 out of 126; 59.52%), regardless of their age category.

An extremely small percentage of respondents (32.54%) declared that they consume supplements on the recommendation of their doctor or pharmacist. Most of them use the supplements on their own initiative (36.5%) or on the advice of a friend. From the group of patients under the age 65 years old, most of the respondents have been consuming supplements for 1 month, while in the other two groups, the most frequently declared duration of the consumption of the supplements was over 2 months. More than half of the users (55.56%) noted major positive changes in their health status after they started consuming the nutritional supplements (Table 4).

### 3.3. Nutritional Status Assessment

Among 65 patients, the average age is 65.81 ± 4.29, comprising 16 men (24.6%) and 49 women (75.4%). The demographic description of the study group is presented in Table 5. The group is parametrically normal.

**Figure 4:**
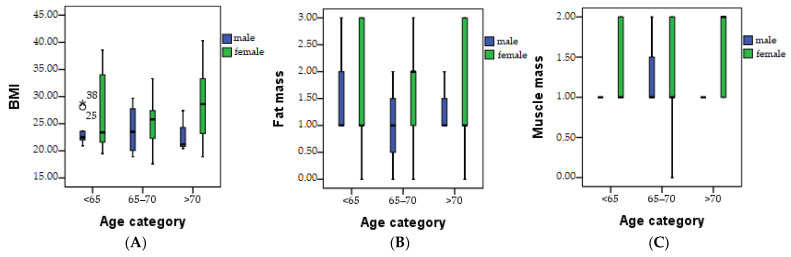

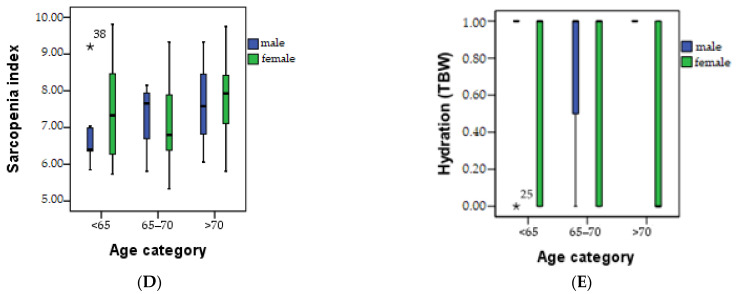
Clinical evaluation of the patients based on BMI (**A**), fat mass (**B**), muscle mass (**C**), sarcopenic index (**D**), and hydration (TBW) (**E**). * mean: statistically significant.

The patients underwent clinical evaluation based on BMI, fat mass, muscle mass, sarcopenic index, and hydration measured, as shown in Figure 4A–E.

An increase in the mean BMI was observed as the age category increased, with the highest values observed in women in the >70 category (Figure 4A). The highest average value of fat mass was observed in the age category of 65–70 for women, as seen in Figure 4B. At the age of 65–70, a normal muscle mass can be obtained, and at over 65, the loss of muscle mass is more pronounced in men. Normal values of the sarcopenic index were observed in the studied patients, with the appearance of a risk in men over the age of 65, as shown in Figure 4D.

In the current study, below normal levels of total water were observed in women above the age of 70, particularly in those with the highest BMI, as shown in Figure 4E.

Seventy-six percent of the patients used nutritional supplements, different types of vitamins, minerals, and plant extracts to prevent osteoporosis, improve immunity, etc. Meanwhile, 35.38% of patients used HP.

**Figure 5:**
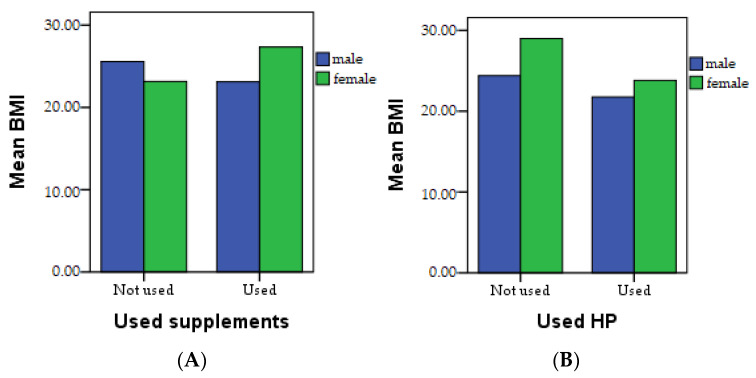

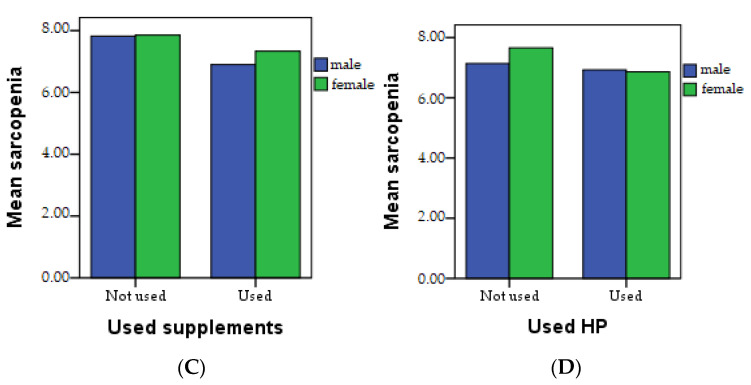
Clinical evaluation of the patients based on BMI and sarcopenia index; (**A**) BMI in patients who do not consume supplements and in patients who consume supplements; (**B**) BMI in patients who consume supplements and in patients who consume HP; (**C**) Sarcopenic index in patients who do not consume supplements and in patients who consume supplements; (**D**) Sarcopenic index in patients who consume supplements and in patients who consume HP.

Figure 5 and Table 6 show the results obtained after comparing the clinical evaluation indicators between the patients who do not consume supplements, patients who consume any type of nutritional supplements, and patients who consume HP. The BMI index, as depicted in Figure 5A,B, demonstrates higher values in patients who consume nutritional supplements compared to patients who do not. This is likely due to a higher consumption of vitamins and minerals that can stimulate appetite. On the contrary, it is noteworthy that patients who use HP exhibit a lower average BMI value. Notably, elevated values of the sarcopenic index were observed in the examined patients, as illustrated in Figure 5C,D.

The level of fat mass is within the normal range for most patients. In 23.8% of patients who use various types of supplements, the fat mass is above the normal range, and in 26.2% of them, it is categorized as high. Among patients using hepatoprotective supplements (HP), it is worth noting that only 21.7% exhibit a level above the normal range, and merely 4.3% have a high level of fat mass.

Concerning muscle mass, most patients demonstrated normal values. Additionally, the hydration level of the body falls within the normal range for most patients. However, among those who consume different types of supplements, we observe low hydration levels in 42.9% of cases. Remarkably, this level drops significantly to 17.4% among patients using HP supplements, as presented in Table 5. It is noteworthy that in the category of women over 70 years who possess a higher body mass index (BMI), there was an observed reduction in the total amount of water below the normal level.

## 4. Discussion

Plants have served multiple purposes over time, not only as a source of food but also as medicinal remedies [37]. Traditionally, people commonly utilized the fruits and roots of plants, often overlooking other parts of the plants.

The liver, acknowledged as a vital organ, performs a variety of essential functions [38]. These functions encompass the production of proteins, cholesterol, and bile, as well as the storage of vitamins, minerals, and carbohydrates [39]. Furthermore, the liver plays a critical role in detoxifying the body by metabolizing substances like alcohol, medications, and metabolic by-products to maintain overall metabolic equilibrium within an organism [40,41]. Consequently, it is particularly vulnerable to oxidative stress [42]. Both the hepatoprotective and antioxidant effects are commonly found together in most hepatoprotective supplements (HP).

A study conducted in 2022 [43] examined the efficacy of hepatoprotective measures in preventing the development of liver lesions resulting from drug treatments. The study revealed a notable effectiveness of these measures with no significant associated health risks. This demonstrates that the current research focuses on addressing a pressing health issue, specifically liver damage, for which the utilization of natural HP may serve as a potential solution.

The results show that more than half of the subjects (76.93%) with chronic diseases who participated in this study use hepatoprotective supplements. A high percentage of patients choose to use supplements based on *Silybum marianum* L. Gaertn., which shows that this is the most known, recommended, and used HP.

Certainly, while the majority of research indicates that individuals with chronic liver conditions often incorporate nutritional supplements into their routines [44], our study specifically examined the utilization of hepatoprotective supplements (HP) in patients who had non-hepatic chronic diseases. The treatment of chronic conditions typically involves a prolonged use of medication. While these drugs are effective in managing the illness, their complete advantages are frequently not fully achieved due to the fact that roughly 50% of patients do not adhere to their prescribed medication regimens [45]. Patients who are prescribed numerous drugs for chronic diseases tend to have lower adherence to preventive hepatoprotective treatments [46].

In this study, the number of prescribed medicines had an impact on how frequently HP was consumed. Group 7 had the highest consumption rates (83.33%, N = 40/48), followed by Groups 3 (82.84%, N = 180/217) and 4 (80.44%, N = 144/179), while Group 1 had the lowest usage rates (58%, N = 36/62). Most of the participants (59.5%) used extracts of *Silybum marianum.*

Even if nutritional supplements have many beneficial effects for health, they should be consumed on the recommendation of a specialist, following a carefully studied scheme, in suitable doses and in such a way as to avoid interactions with the drugs used at the same time. To see the supplement consumption habits among the patients with chronic diseases included in the study, a simple and reliable questionnaire was developed. This monitored whether the patient’s used supplements and, if so, the reasons for use (hepatoprotection and prevention, among others). The study also tracked where the supplements were purchased (pharmacy, naturists, internet, or others), who recommended them (own initiative, friends, mass media, doctor’s advice, and pharmacist’s advice, among others), whether the recommended dose was respected, and the duration of supplement usage.

It was found that most patients use nutritional supplement on their own initiative or obtain information from the mass media. Only a small proportion of them (32.54%) use HP on the recommendation of the doctor or pharmacist, which corresponds to the tendency of self-medication of patients, which is observed worldwide. A percentage of 54% of patients have been using supplements for two months or longer.

Regarding nutritional status, a percentage of 88% of patients who use supplements showed minor or major improvements regarding their health status, compared to the percentage of 7.92% of patients who did not notice improvements in their health status.

The clinical evaluations to which the patients underwent were based on the measurement of BMI, fat mass, muscle mass, sarcopenic index, and hydration measurement. It was found that patients using HP have a lower mean BMI value, both compared to patients who use other types of nutritional supplements and compared to patients who do not use supplements. High values of the sarcopenic index were observed in the studied patients, which is associated with the infiltration of fats between actin and myosin, which can block insulin receptors and correlate with metabolic syndrome and other metabolic diseases. As the fat mass increases, the degree of hydration of the body is lower, triggering a pro-inflammatory process with water retention in the intercellular spaces. Among the patients who use HP, it can be observed that only 21.7% have a level above normal and only 4.3% have a high level of fat mass. Regarding hydration status, low levels can be observed in patients who consume different types of supplements. However, in patients who consume HP supplements, this level is much lower.

**Figure 6:**
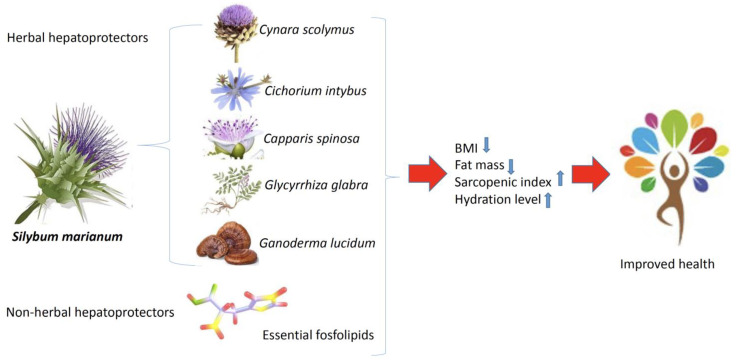
Most relevant outcomes summarized.

The likely mechanisms of action for these herbs may involve immunomodulation and a stimulation of hepatic DNA synthesis, as well as the stimulation of superoxide dismutase and glutathione reductase to inhibit oxidative stress in hepatocytes. Additionally, they can help reduce intracellular reactive oxygen species by enhancing antioxidant levels [47]. In our study, individuals who integrate nutritional supplements into their daily routines typically observe enhanced well-being. This improvement in health is associated with the sustained use of hepatoprotective supplements (HP) for a period exceeding 2 months. Most important outcomes of our research can be schematized, as is depicted in the Figure 6.

Due to the limitations of the sample (particularly, sampling methodology, sociodemographic characteristics, and sample size), it may not be viable to generalize our findings to the Romanian population.

It would be necessary to recruit more participants, to create a more age and gender-heterogeneous cohort for which to evaluate more precisely the chronic disease and the type/duration of the treatment, for providing more accurate results.

However, it is worth emphasizing that for the population with chronic diseases in Romania, until now, no similar study has been conducted regarding the use of HP. Therefore, this study represents a reference, and it allows similar application to other age groups, other disease categories, or to the general population.

## 5. Conclusions

The present study highlights that a majority of patients with chronic diseases utilize hepatoprotective nutritional supplements. In most cases, patients either initiate the use of HP themselves or acquire information from mass media sources. Patients who incorporate nutritional supplements into their routines generally experience improved health. This enhancement in health is linked to the utilization of HP for a duration exceeding 2 months. Furthermore, these advancements in health status were substantiated by outcomes derived from clinical analyses, encompassing BMI, fat mass, sarcopenic index, and hydration level.

## Figures and Tables

**Table 1:** Study group description.

Groups	N	%
1	62	6.5
2	217	22.7
3	217	22.7
4	179	18.8
5	139	14.6
6	92	9.6
7	48	5

N, number of patients; 1–7, number of the group/medicines for chronic conditions.

**Table 2:** The HP distribution in patients by the groups.

Groups	No		*Silybum*		Other Types of HP	
	Count %					
1	26	12.0	29	5.1	7	4.1
2	53	24.4	137	24.1	27	16.0
3	37	17.1	122	21.5	58	34.3
4	35	16.1	117	20.6	27	16.0
5	38	17.5	76	13.4	25	14.8
6	20	9.2	57	10.0	15	8.9
7	8	3.7	30	5.3	10	5.9
Total	217	22.7	568	59.5	169	17.7

Legend: No—patients who do not use HP; Silybum—patients using HP based on *Silybum marianum* L. Gaertn; other types of HP—patients using another type of supplement based on *Cichorium intybus* L., *Armoracia rusticana* L., *Carum carvi* L., *Curcuma longa* L., *Piper nigrum* L., *Capparis spinosa* L., *Glycyrrhiza glabra* L., *Ganoderma lucidum* Kanst., *Chlorella* spp., *Spirulina* spp., soybean seeds, Indian currant extracts, essential phospholipids, L-arginine.

**Table 3:** Demographic description of the study group.

Parameters		Age Category						*p*
		<65		65–70		>70		
		N %						
Sex	Male	27	13.8	12	6.2	9	4.6	0.001
	Female	69	35.4	51	26.2	27	13.8	
Origin	Urban	69	35.4	51	26.2	18	9.2	0.433
	Rural	27	13.8	12	6.2	18	9.2	
Education	Secondary	51	26.2	33	16.9	15	7.7	0.004
	Higher	45	23.1	30	15.4	21	10.8	
Total		96	49,2	63	32.3	36	18.5	0.002

**Table 4:** Results obtained after the evaluation of the study groups by questionnaire.

Questions	Answer Option	Age Category						*p*
		<65		65–70		>70		
		N %						
Use of supplements	No	33	16.9	21	10.8	15	7.7	0.017 *
	Yes	63	32.3	42	21.5	21	10.8	
For what purpose?	No	33	16.9	21	10.8	15	7.7	0.042 *
	Hepatoprotective	32	16.41	28	14.35	15	7.7	
	Body protection	18	9.23	3	1.53	0	0.0	
	Preventive	7	3.58	2	1.02	0	0.0	
	Other	6	3.07	9	4.61	6	3.07	
Recommended by	Don’t use	33	16.9	21	10.8	15	7.7	0.096
	Their own initiative	24	12.3	16	8.20	6	3.07	
	Friends	9	4.61	3	1.53	0	0.0	
	Mass media	12	6.15	6	3.07	3	1.5	
	Doctor	9	4.61	9	4.6	6	3.1	
	Pharmacist	6	3.07	5	2.56	6	3.07	
	Other	3	1.53	3	1.53	0	0.0	
How long	Don’t use	33	16.9	21	10.8	15	7.7	0.312
	Not yet	0	0.0	3	1.53	0	0.0	
	Less than a week	12	6.15	6	3.07	0	0.0	
	1–2 weeks	13	6.66	2	1.02	0	0.0	
	1 month	17	8.71	5	2.56	0	0.0	
	2 months	6	3.07	8	4.10	6	3.07	
	More than 2 months	15	7.7	18	9.23	15	7.7	
Changes in health status	Don’t use	33	16.9	21	10.8	15	7.7	0.744
	Major	35	17.94	26	13.33	9	4.61	
	Minor	17	8.71	15	7.7	9	4.61	
	No change	6	3.07	1	0.51	3	1.53	
	Worsening	0	0.0	0	0.0	0	0.0	

N, number of patients; *p*, statistical significance; * mean statistically significant

**Table 5:** Demographic description of the study group.

Parameters		Age Category						*p*
		<65		65–70		>70		
		N %						
Sex	male	9	13.8	4	6.2	3	4.6	0.763
	female	23	35.4	17	26.2	9	13.8	
Origin	urban	23	35.4	17	26.2	6	9.2	0.173
	rural	9	13.8	4	6.2	6	9.2	
Education	secondary	17	26.2	11	16.9	5	7.7	0.791
	higher	15	23.1	10	15.4	7	10.8	
Total		32	49.23	21	32.30	12	18.46	0.356

N, number of patients; *p*, statistical significance.

**Table 6:** Clinical evaluation of the patients based on fat mass, muscle mass, hydration.

Evaluated Parameters		Used Supplements		Used HP		*p*
		Not Used	Used	Not Used	Used	
		N(%)				
Fat mass	under	1(16.7)	6(10.2)	2(4.8)	5(21.7)	0.117
	normal	3(50.0)	28(47.5)	19(45.2)	12(52.2)	0.134
	over	2(33.3)	13(22.0)	10(23.8)	5(21.7)	0.219
	high	0(0.0)	12(20.3)	11(26.2)	1(4.3)	1.000
Muscle mass	under	0(0.0)	1(1.7)	0(0.0)	1(4.3)	1.000
	normal	5(83.3)	39(66.1)	23(54.8)	21(91.3)	0.025 *
	over	1(16.7)	19(3.,2)	19(45.2)	1(4.3)	0.819
Hydration	under	1(16.7)	21(35.6)	18(42.9)	4(17.4)	0.637
	normal	5(83.3)	38(64.4)	24(57.1)	19(82.6)	0.036 *
	high	0(0.0)	0(0.0)	0(0.0)	0(0.0)	1.000

N, number of patients; *p*, statistical significance; * mean statistically significant

## Data Availability

Data are available at the first author per request.

## Supplementary Materials

The following supporting information can be downloaded at: https://www.mdpi.com/article/10.3390/healthcare11192685/s1, Supplementary Material S1 (Questionnaire).
