# Supplementary material for: Highlighting the Use of the Hepatoprotective Nutritional Supplements among Patients with Chronic Diseases
Source: Healthcare (Basel). 2023 Oct 6;11(19):2685. doi: 10.3390/healthcare11192685 (PMC10572698; doi:10.3390/healthcare11192685)
Supplement: Supplementary file 1 [file healthcare-11-02685-s001.zip › healthcare-2607748-supplementary.pdf]

Supplementary material S1

QUESTIONNAIRE

Dear Sir/Madam, with this questionnaire we invite you to participate in a study on the administration of dietary supplements.

Your answers are important for us. Thank you!

Age.......................................

Gender F___, M___

Environment of origin Urban ☐

Rural ☐

Do you use dietary supplements? YES ☐

NO ☐

If yes, in which case?

Liver disease		Protection of the organism	
Prevention		Others	

What type of dietary supplement do you use ? ……………………………………………………………………………

The use of the dietary supplement was:

Own initiative		Friends' advice		Mass media	
The doctor's advice		Pharmacist's advice		Others	

Regarding the administered dose

I use the recommended dose in the supplement leaflet		I use the dose recommended by the doctor/pharmacist	
I use a higher dose than the recommended dose		I use a lower dose than the recommended dose	

How long have you been using the dietary supplement?

I haven't started yet		Less than a week		1-2 weeks	
One month		Two months		More than two months	

During the period of using dietary supplements, what changes have you noticed in your health status?

Major improvements		Minor improvements	
No change		Worsening	
